# Supplementary material for: Meeting demand—Obstetric hemorrhage and blood availability in Malawi, a qualitative study
Source: PLoS One. 2022 Aug 24;17(8):e0273426. doi: 10.1371/journal.pone.0273426 (PMC9401179; doi:10.1371/journal.pone.0273426)
Supplement: Attachment — Submitted filename: Response To Reviewers.docx [file pone.0273426.s002.docx]

Journal Requirements:

1. We have revised our manuscript to meet PLOS ONE’s style requirements.
2. Due to ethical restrictions, we are unable to share a de-identified data set as the data contains potentially sensitive information that is pertinent to the topic at hand, thus redaction of said information would result in a loss of context. These restrictions were put in place by the College of Medicine Research and Ethics Committee (COMREC) at the Kamuzu University of Health Sciences (previously known as the University of Malawi, College of Medicine). The research team is happy to provide a minimally anonymized data set on reasonable request, with requests to be sent to COMREC at the following address:

IRB Administrator

College of Medicine Research and Ethics Committee (COMREC)

Kamuzu University of Health Sciences (KUHES)

[comrecadmin@medcol.mw](mailto:comrecadmin@medcol.mw)

**Editor/Reviewer Comments:**

(Note: All of the revisions and specific line numbers referenced below are based on the “Revised Manuscript with Track Changes” document for efficiency of review)

1. The study appears to present the results of original research. I agree with the reviewer that citing prior work from other jurisdictions, or systematic reviews, would be helpful where available.

Thank you for this suggestion. We have added additional citations, where available, that provide more situational context.

2. Results do not appear to have been published elsewhere.

Confirming that we have not published these results elsewhere.

3. Experiments, statistics, and other analyses are performed to a high technical standard and are described in sufficient detail. Thank you.

4. Conclusions are presented in an appropriate fashion and are supported by the data. Thank you.

5. The article is presented in an intelligible fashion and is written in standard English. Thank you.

6. The authors should clarify whether their institutional review board has granted permission to identify participants by their position description. This would appear to make these people identifiable, which may be allowed, but I think the journal would want to clarify this.

Thank you for bringing this critical point to our attention. We can confirm that the institutional review board has granted permission to identify participants by their position description, with a few exceptions for individuals whose job descriptions make these people identifiable. These job descriptions were revised accordingly (lines 246-247, 382).

7. The article adheres to appropriate reporting guidelines and community standards for data availability. In saying that, I think the study report could benefit from checking compliance with an appropriate reporting checklist such as COREQ or SRQR.

Thank you for suggesting SRQR. We did indeed utilize this checklist as we drafted the manuscript. We have included the checklist in the supplemental materials (note: page and line numbers in the checklist correspond to the final, unmarked “Manuscript” document).

Background
Page 2(line 42): Authors might want to search for (and add) some recent systematic analyses, if available

Thank you for this suggestion. We have revised the document to reference the most recent systematic analyses available.

Methods

Page 4 (line 91) : Have you given the abbreviation for SN and AM somewhere before ? If not, better to give the abbreviation here.

Thank you for this suggestion. We have clarified that these abbreviations refer to author initials in the parentheses (line 111).

Page 5 (line 96) : How was the interview guide revised based on the pretest? What were the major adjustments done on interview guide based on the pre-test results?

Thank you for this question. The major adjustments done to the interview guide based on pre-test results include the removal of questions that lacked clarity and resulted in responses that did not contribute to the data in a meaningful way. This has been further clarified in line 118-119.

Page 5 (lines 101 - 103) : Recheck for the punctuations. I think the 'comma' is required after QECH. The line should go like - Queen Elizabeth Central Hospital (QECH), a large urban referral hospital; Mulanje……………………………………………………..

Thank you for this suggestion. We have edited this line (now 123-125) to include proper punctuations.

Page 8 (line 153) : How was the interview guide standardized?

Thank you for this question. The interview guide included a fixed set of questions that were asked of all participating subjects. Additional follow up questions were asked for clarity and further elaboration as needed. This has been clarified in lines 176-178.

Page 9 (line 183) : Was it the written consent, better to specify.

Thank you for this suggestion, specified in line 210.

Results

Overall: Result section focuses on describing the barriers and challenges encountered around five different themes. The solutions/recommendations are given in bullets in table only, not described adequately. I think term used in the title- 'meeting demand' indicates the need of equal weightage to the solution part also. Therefore, I suggest some more elaboration on solutions to barriers/ challenges under each theme.

Thank you for calling our attention to the discrepancy between what is reported in the table and in the text. We agree that the solutions deserve more description and have added more detail in the text regarding the recommended solutions in lines 441-481.

Page 10 (line 201): What are the societal norms hindering blood donation? The description given below this line specify the individual preferences whether to donate blood or not, but it is not capturing the barriers related to societal norms as a whole. Authors might want to recheck or rethink on use of term 'societal norms' here/ or some descriptions on 'societal norms' can be added (if data are available).

Thank you for this question. In this case, rather than societal norms per say, it is that the general population is still operating under the principles of the pre-existing methods of blood donation and collection, that is the principle of family member donations at the hospital. This was acknowledged by several subjects as a barrier to voluntary blood donations, as many noted that people often state that they would only need to donate if a family member is in need. The language has been modified to better reflect this concept in lines 229-232.

Discussion -
Reads very well.
I would prefer adding one paragraph on strengths, and generalizability of findings in other settings.

We have added a few lines (559-563) regarding the generalizability of findings to the end of the discussion section. In this paragraph, we referenced studies that have also sought to assess challenges with and opportunities to improve access and distribution of blood products in other settings.
